# Breaking down Leukemia Walls: Heteronemin, a Sesterterpene Derivative, Induces Apoptosis in Leukemia Molt4 Cells through Oxidative Stress, Mitochondrial Dysfunction and Induction of Talin Expression

**DOI:** 10.3390/md16060212

**Published:** 2018-06-17

**Authors:** Yu-Cheng Chen, Mei-Chin Lu, Mohamed El-Shazly, Kuei-Hung Lai, Tung-Ying Wu, Yu-Ming Hsu, Yi-Lun Lee, Yi-Chang Liu

**Affiliations:** 1 The Ph.D. Program for Cancer Biology and Drug Discovery, China Medical University and Academia Sinica, Taichung 404, Taiwan; j520c1@gmail.com; 2 Graduate Institute of Marine Biotechnology, National Dong Hwa University, Pingtung 944, Taiwan; jinx6609@nmmba.gov.tw; 3 National Museum of Marine Biology & Aquarium, Pingtung 944, Taiwan; mos19880822@gmail.com; 4 Department of Pharmacognosy, Faculty of Pharmacy, Ain-Shams University, Organization of African Unity Street, Abassia, Cairo 11566, Egypt; mohamed.elshazly@pharma.asu.edu.eg; 5 Department of Pharmaceutical Biology, Faculty of Pharmacy and Biotechnology, German University in Cairo, Cairo 11432, Egypt; 6 Graduate Institute of Natural Products, College of Pharmacy, Kaohsiung Medical University, Kaohsiung 807, Taiwan; kuma0401@gmail.com; 7 Research Center for Environmental Medicine, Kaohsiung Medical University, Kaohsiung 807, Taiwan; maiz538272@gmail.com; 8 Department of Urology, Sinying Hospital, Ministry of Health and Welfare, Tainan 730, Taiwan; leeyl05@yahoo.com.tw; 9 Division of Hematology-Oncology, Department of Internal Medicine, Kaohsiung Medical University Hospital, Kaohsiung 807, Taiwan; 10 Department of Internal Medicine, Faculty of Medicine, College of Medicine, Kaohsiung Medical University, Kaohsiung 807, Taiwan

**Keywords:** heteronemin, reactive oxygen species, mitochondrial, talin, phosphorylated talin

## Abstract

Heteronemin, the most abundant secondary metabolite in the sponge *Hippospongia* sp., exhibited potent cytotoxic activity against several cancer cell lines. It increased the percentage of apoptotic cells and reactive oxygen species (ROS) in Molt4 cells. The use of ROS scavenger, *N*-acetyl cysteine (NAC), suppressed both the production of ROS from mitochondria and cell apoptosis that were induced by heteronemin treatment. Heteronemin upregulated talin and phosphorylated talin expression in Molt4 cells but it only upregulated the expression of phosphorylated talin in HEK293 cells. However, pretreatment with NAC reversed these effects. Talin siRNA reversed the activation of pro-apoptotic cleaved caspases 3 and 9. On the other hand, the downstream proteins including FAK and NF-κB (p65) were not affected. In addition, we confirmed that heteronemin directly modulated phosphorylated talin expression through ROS generation resulting in cell apoptosis, but it did not affect talin/FAK complex. Furthermore, heteronemin interfered with actin microfilament and caused morphology changes. Taken together, these findings suggest that the cytotoxic effect of heteronemin is associated with oxidative stress and induction of phosphorylated talin expression. Our results suggest that heteronemin represents an interesting candidate which can be further developed as a drug lead against leukemia.

## 1. Introduction

Mitochondria, the cellular powerhouse, play an important role in ATP (adenosine triphosphate) production and in cellular apoptotic/antiapoptotic balance [1,2]. The interruption of ATP production blocks macromolecules’ synthetic machinery and this process is essential for cellular proliferation. Therefore, mitochondrial ATP production represents an interesting avenue in the war against cancer through halting the proliferation of cancer cells [3]. ATP production is accompanied by ROS (reactive oxygen species) generation in normally functional mitochondria, due to electron leakage from the respiratory chain and semiquinone radicals generated at complexes I, II and III. ROS can be converted to H_2_O_2_ by superoxide dismutase 2 (SOD2) which can be further converted to H_2_O by glutathione peroxidase [4,5,6]. It was found that ROS levels could also affect cellular proliferation. Escalated ROS production along with continued mitochondrial permeability transition pore (mPTP) openings may lead to elevated ROS levels resulting in the reduction of mitochondrial membrane potential (MMP), induction of cytochrome *c* release, accumulation of proapoptotic protein (BAX), activation of caspases and cell death [7]. Several reports indicated that ROS may intervene with cell signaling pathway including MAPK, NF-κB and AKT pathways that dominate cancer cells proliferation and survival [8,9]. Moreover, elevated levels of ROS can inhibit energy production in cancer cells resulting in cancer cells death [6]. The capacity of energy production and oxidative balance render mitochondria as attractive target in cancer treatment.

Another potential target affecting cancer cells proliferation, which attracted attention in the last two decades, is a cytoskeletal protein with 2541 amino acids and molecular mass 270 KDa. This protein, talin, plays a significant role in integrin activation mediated cell adhesion, migration, and proliferation. It is also a focal adhesion player that binds to integrin, vinculin, focal adhesion kinase capacity (FAK) and actin [10,11]. It was found that FAK is activated when talin binds to integrin and promotes capacity cell survival and proliferation through protein kinase B (AKT), NF-κB and ERK survival pathways [12]. Recent reports indicated that the serine threonine kinase AKT is constitutively activated in 70–85% of T-ALL (T-acute lymphoblastic leukemia) patients and 38% of the cases show an up-regulation of ERK [13]. AKT is also involved in many tumor-associated cellular regulation mechanisms such as promoting cell growth, survival, and angiogenesis [14]. Recent studies demonstrated that talin is an oncogene-associated protein in breast, prostate and liver cancers [15,16,17]. Certain compounds such as the marine toxin bistratene A were found to target talin by inducing its phosphorylation causing morphological changes [18]. However, limited information is known about the consequences of talin phosphorylation in cancer cells. This study suggests that talin phosphorylation mediates apoptosis in cancer cells and serves as a tumor suppressor gene.

Marine environment forms the richest ecological system on earth with millions of species living together in a continuous process of interaction and competition. Sponges, corals, ascidiacea and marine microorganisms survived for millions of years through complex adaptation processes. Among these processes was the development of sophisticated biosynthetic machinery to produce secondary metabolites which can deter and kill predators at extremely high dilution rendering them excellent potential cytotoxic candidates. Specific classes of secondary metabolites showed a certain type of exclusivity to marine organisms and exhibited potent cytotoxic activity including sesterterpenoids. This group of terpenoids comprises less than 1000 known compounds which can be classified based on their carbocycle numbers into six subgroups including linear, monocarbocyclic, bicarbocyclic, tricarbocyclic, tetracarbocyclic, and miscellaneous sesterterpenoids [19]. These compounds showed interesting biological effects such as antimicrobial, antifeedant, anti-inflammatory and cytotoxic activities [19].

The challenging chemical structures and potent biological activities of sesterterpenoids encouraged us to pursue a detailed investigation of their presence in Asian marine sponges. Aiming to find new apoptotic secondary metabolites, we isolated a sesterterpenoid derivative, heteronemin, from the marine sponge of *Hippospongia* sp. and discovered its potent cytotoxicity against human carcinoma cell lines with IC_50_ < 0.001 μg/mL after 72 h [20]. The same sesterterpenoid derivative was also isolated from another sponge, *Hyrtios* sp., and exhibited potent cytotoxic activity against A498 human renal carcinoma cells through the disruption of mitochondrial function. The search for heteronemin molecular targets indicated that this sesterterpenoid affects TDP-43, which is a key factor in neurodegenerative disorders. Heteronemin also inhibited TNF-α induced NF-κB activation through proteasome inhibition [21,22]. These findings highlight the importance of heteronemin as a promising cytotoxic candidate. However, previous reports did not investigate heteronemin cytotoxic mechanism of action against human acute lymphoblastic leukemia cells. In the current study, we investigated the effect of heteronemin on ROS generation and talin expression. A correlation was established between the effect of heteronemin on these molecular targets and its apoptotic activity against human acute lymphoblastic leukemia cells.

## 2. Results

### 2.1. Cytotoxic Activity of Heteronemin against Different Cancer Cell Lines and Its Apoptotic Induction Activity against Molt4 Cells

**Figure 1:**
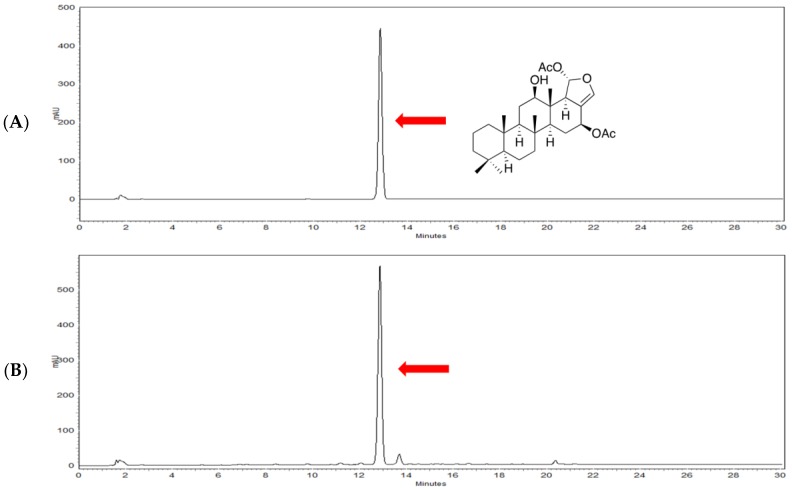

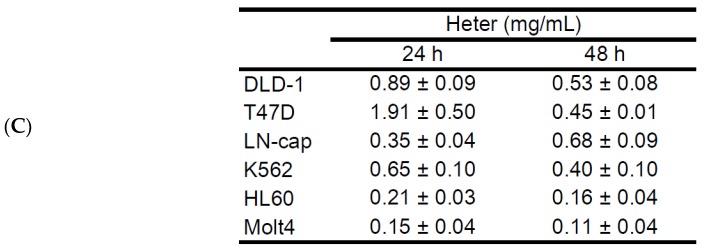
HPLC chromatogram of the isolated heteronemin (**A**) and extracts (**B**) of *Hippospongia* sp. for comparison as well as cytotoxic activity against human cancer cell lines (**C**). IC_50_ values were calculated by calcuSyn software (Biosoft, Ferguson, MO, USA). Results are shown as mean ± SD of three independent experiments.

To fully reveal the potential application of heteronemin as a promising secondary metabolite, we evaluated its concentration in *Hippospongia* sp. sample. Heteronemin which was isolated from our previous study was regarded as the standard compound and it was co-eluted with *Hippospongia* sp. extract. HPLC analysis indicated that the concentration of the heteronemin was 621.56 μg in 1.0742 g of *Hippospongia* sp. sample suggesting 58% of extraction yield (Figure 1A,B). After demonstrating the richness of *Hippospongia* sp. sample with heteronemin, we then moved to determine its IC_50_ values against numerous cancer cell lines such as colon (DLD-1), breast (T47D), prostate (LN-cap) and leukemia cell lines (K562, HL60, and Molt4) for 24 and 48 h. After 48 h, leukemia cell lines were more sensitive to the cytotoxic effect of heteronemin showing IC_50_ values of 0.41 ± 0.08 for K562, 0.16 ± 0.05 for HL60 and 0.10 ± 0.04 for Molt4 as demonstrated by the MTT assay (Figure 1C).

**Figure 2:**
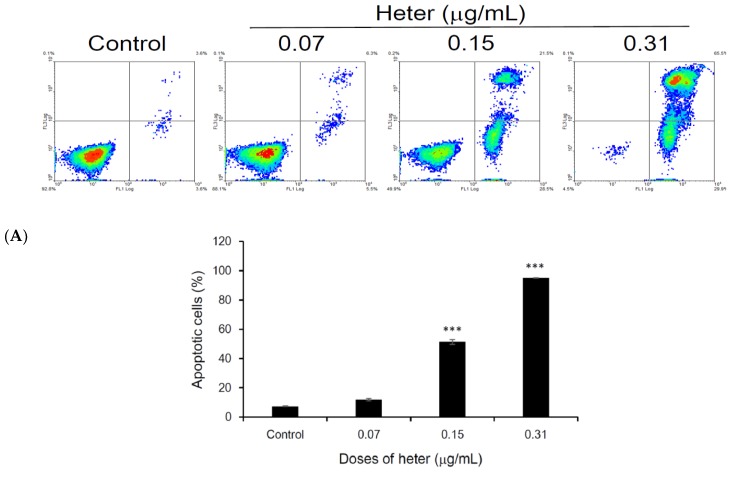

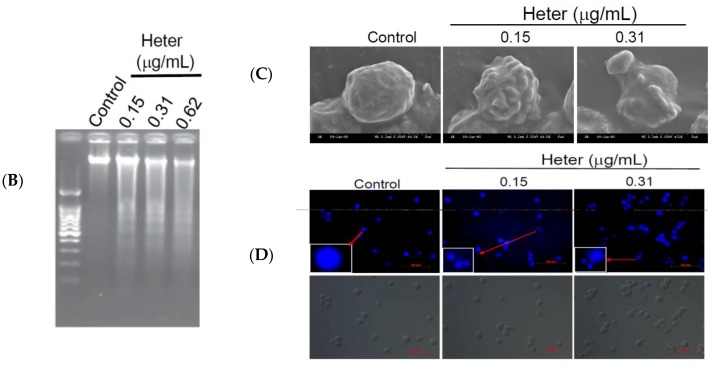
Heteronemin induces apoptosis in Molt4 cells. (**A**) Double staining with annexin V/propidium iodide (PI) was used to clarify the apoptotic induction following the treatment of Molt4 cells with heteronemin (0.07, 0.15 and 0.31 μg/mL) for 24 h. Results are presented as mean ± SD of three independent experiments, (*** *p* < 0.001); (**B**) DNA fragmentation was determined with electrophoresis; (**C**,**D**) Morphological changes were detected via examination by staining with SEM (9k ×; up panel) and DAPI (200 ×).

The potent cytotoxic activity of heteronemin encouraged us to investigate its mechanism of action against Molt4 cells. First, we evaluated whether the growth inhibitory activity of heteronemin against Molt4 cells is associated with apoptosis using annexin-V-FITC and propidium iodide (PI) assay. As shown in Figure 2A, treating Molt4 cells with heteronemin at 0.07, 0.15 and 0.31 μg/mL increased the percentage of apoptotic cells (annexin V-FITC positive cell) from 3.7% to 14.7%, 63%, and 98.1%, respectively. Furthermore, we evaluated the effect of heteronemin on DNA and found that it triggered DNA fragmentation (100~300 kbp) from 0.15 μg/mL to 0.62 μg/mL (Figure 2B). Morphological analysis using scanning electron microscope showed wrinkles and blisters upon treating Molt4 cells with heteronemin (0.15 and 0.31 μg/mL) compared with the control which demonstrated integral cells with clear surfaces (Figure 2C). Also, nuclear condensation and apoptotic bodies were observed after treating Molt4 cells with heteronemin (0.15 and 0.31 μg/mL) as demonstrated by DAPI staining (Figure 2D).

### 2.2. Effect of Heteronemin on Tumor Growth In Vivo Human Molt4 Xenograft Animal Model

**Figure 3:**
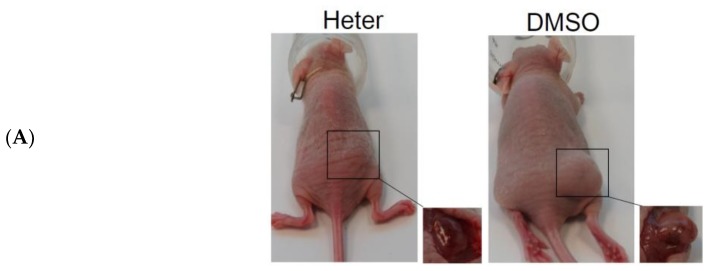

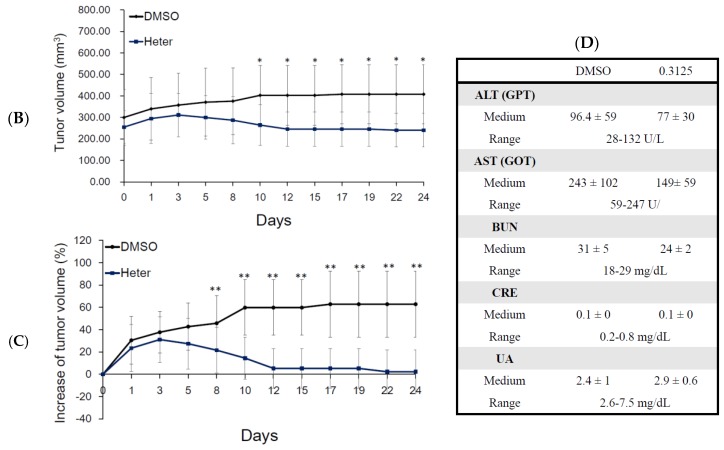
Antitumor activity of heteronemin on tumor growth in vivo human Molt4 xenograft animal model. Tumor-bearing nude mice were treated with solvent (negative control) and heteronemin (0.31 μg/g) for 29 days (each group contained six mice). (**A**) Photos of tumor growth in xenograft nude mice that received solvent only (up) and heteronemin (down); (**B**) Quantitative results show a gradual decrease in tumor volume with heteronemin treatment when compared with the control (DMSO); Results are presented as mean ± SD of three independent experiments, (* *p* < 0.05); (**C**) Effect of heteronemin on the growth of tumor volume (%); The curve of tumor growth was normalized to staring volume. Results are presented as mean ± SD of three independent experiments, (* *p* < 0.05; ** *p* < 0.01); (**D**) Chemical profiles of plasma were determined with FUJIILM colorimetric analyzer (DRI-Chem 3000, Fujifilm, Minato-Ku, Tokyo, Japan).

To further evaluate the anti-tumor activity of heteronemin, we used a xenograft nude mice model inoculated with Molt4 cells. After tumor formation for 300 mm^3^, the control group was treated with DMSO (100 μL) while the treatment group was injected with heteronemin (0.31 μg/g) (Figure 3A). Our results indicate that heteronemin effectively decreased tumor volume from 502 ± 261 mm^3^ to 222 ± 101 mm^3^ (Figure 3B). Furthermore, the 60% increase in tumor volume was attenuated by heteronemin treatment (Figure 3C). We further determined the plasma indices of liver and kidney functions after heteronemin treatment and the results indicate that there were no significant differences between heteronemin treated group and the control (Figure 3D). These results show that heteronemin effectively reduced tumor growth in vivo without any significant side effects.

### 2.3. Effect of Heteronemin on Mitochondrial Membrane Potential (MMP) and Reactive Oxygen Species (ROS) Levels

**Figure 4:**
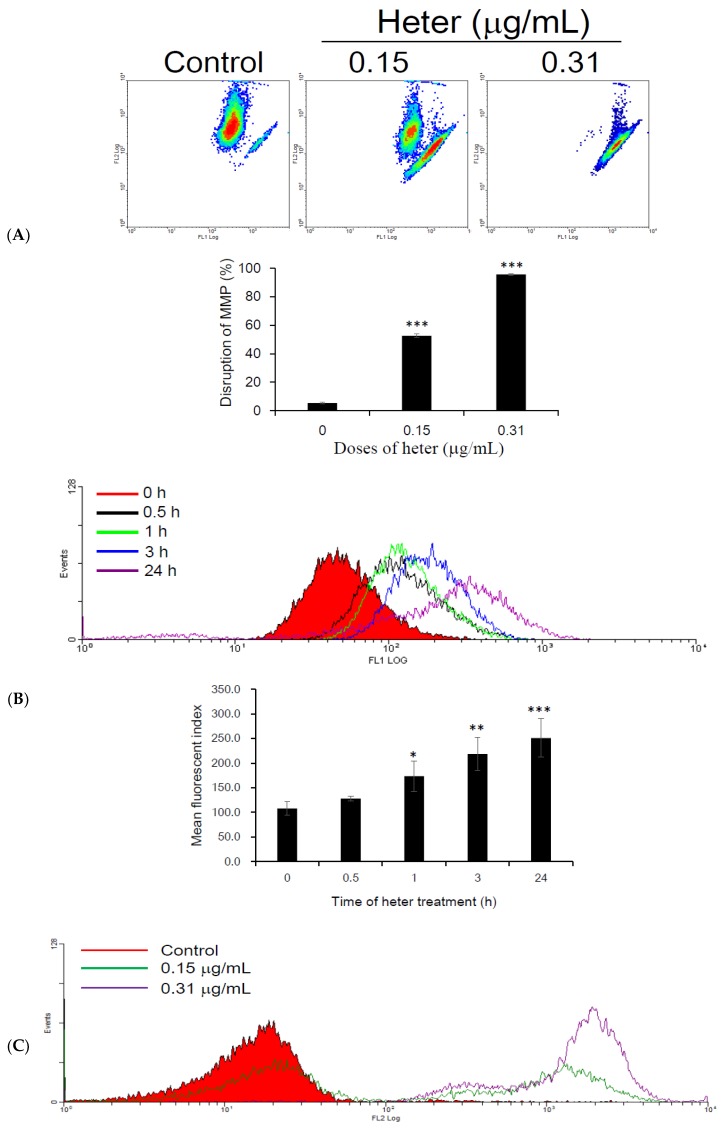

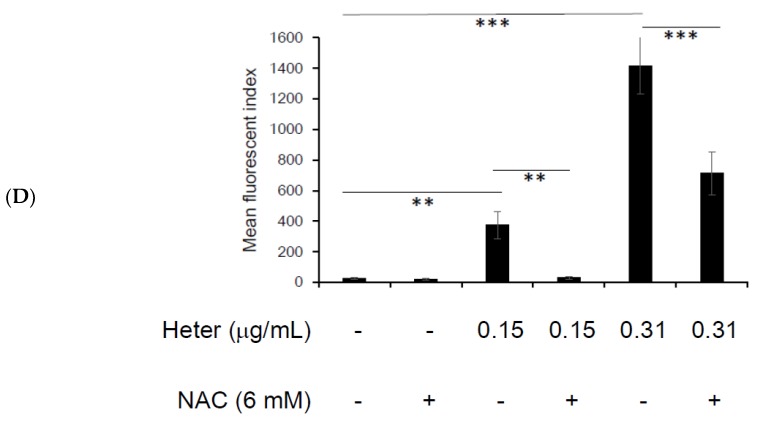
Effect of heteronemin on ROS generation and mitochondrial membrane potential. (**A**) Molt4 cells were treated with different concentrations (0.15 and 0.31 μg/mL) of heteronemin causing disruption of mitochondrial membrane potential (MMP). Results are presented as mean ± SD of three independent experiments, (*** *p* < 0.001); (**B**) Total ROS generation in Molt4 cells after treatment with heteronemin 0.31 μg/mL for the indicated time intervals (0.5, 1, 3 and 24 h). Results are presented as mean ± SD of three independent experiments, (* *p* < 0.05; ** *p* < 0.01; *** *p* < 0.001); (**C**) Mitochondrial superoxide production after treatment with heteronemin 0.15 and 0.31 μg/mL; (**D**) Quantitative results of mitochondrial superoxide production after pretreatment with 6 mM NAC for 2 h. Results are presented as mean ± SD of three independent experiments, (** *p* < 0.01; *** *p* < 0.001).

To evaluate the effect of heteronemin on the integrity of mitochondria in Molt4 cells, we examined mitochondrial membrane potential (MMP) using JC-1 staining and flow cytometry after exposing the cells to heteronemin (0.15 and 0.31 μg/mL) for 24 h. The results reveal that heteronemin treatment disrupted mitochondrial membrane potential in a dose-dependent manner. Quantization results indicate that 0.15 and 0.31 μg/mL of heteronemin decreased mitochondrial membrane potential by 52.5 ± 1.27% and 95 ± 0.57%, respectively (*p* < 0.001) (Figure 4A). These results suggest that the disruption in mitochondrial membrane potential contributed to the heteronemin-induced Molt4 cells apoptosis. The effect of heteronemin on ROS production by Molt4 cells mitochondria was also examined. We determined ROS production with a carboxyl derivative of fluorescein, carboxy-H_2_DCFDA. The results show that the treatment with heteronemin at 0.31 μg/mL for 0.5, 1, 3 and 24 h resulted in 1.04-, 1.12-, 1.33-, 1.79-folds increase in ROS level, respectively as compared with the mean fluorescence index (MFI) of the control (Figure 4B). We further detected the mitochondrial superoxide production after heteronemin 0.15 and 0.31 μg/mL treatment using MitoSOX Red, which is a highly selective superoxide indicator. The results indicate that heteronemin increased the basal level of superoxide production in living cells by 14.98- and 56.65-folds compared with the control (Figure 4C). The above observations suggested that ROS may also contribute to heteronemin induced cell apoptosis.

### 2.4. The Effect of Pretreatment with N-Acetyl Cysteine (NAC) on Heteronemin Induced Apoptosis 

To further clarify whether ROS generation is a major regulator of heteronemin-induced apoptosis, the pretreatment with ROS scavenger, *N*-acetyl cysteine (NAC), was used to suppress intracellular oxidative stress. As shown in Figure 4D, the pretreatment with 6 mM NAC significantly suppressed mitochondrial superoxide generation. These results indicate that heteronemin could induce accumulation of ROS in Molt4 cells and the ROS scavenger, NAC, could suppress such accumulation.

**Figure 5:**
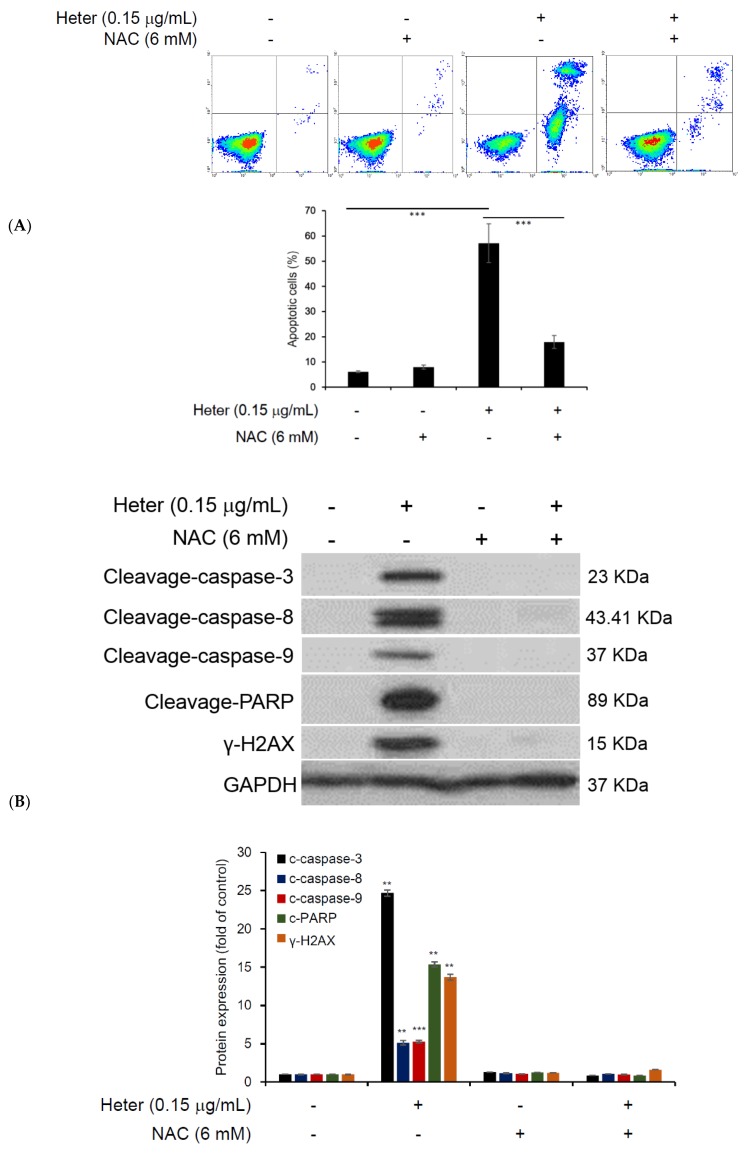

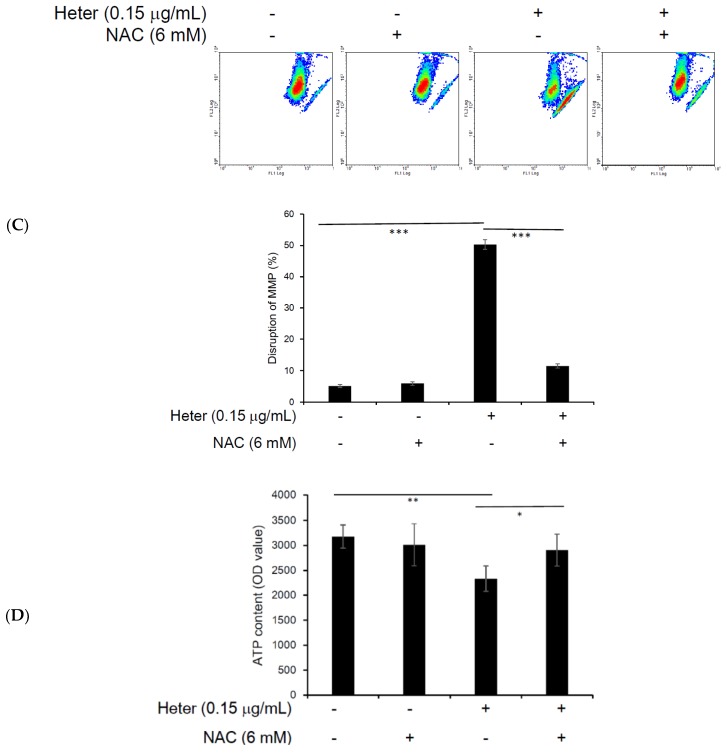
Effect of the pretreatment with NAC, an ROS scavenger, on heteronemin induced apoptosis in Molt4 cells. Cells were pretreated with NAC for 2 h followed by heteronemin (0.15 μg/mL) for 24 h; (**A**) Cells were collected double stained with annexin V/propidium iodide (PI) and analyzed with flow cytometry. Results are presented as mean ± SD of three independent experiments, (*** *p* < 0.001); (**B**) Pro-apoptotic proteins were determined by Western blot assay, GAPDH was used as the loading control; Results are presented as mean ± SD of three independent experiments (** *p* < 0.01; *** *p* < 0.001); (**C**) Disruption of mitochondrial membrane potential in Molt4 cells; Results are presented as mean ± SD of three independent experiments, (*** *p* < 0.001); (**D**) ATP content was determined using ATP determine assay kit after treatment with heteronemin (0.15 μg/mL) for 24 h. Results are presented as mean ± SD of three independent experiments (* *p* < 0.05; ** *p* < 0.01).

To further determine whether heteronemin-induced apoptosis was dependent on ROS overproduction, Molt4 cells were cultured in the presence or absence of ROS scavenger, NAC, for 2 h and then analyzed by annexin V/propidium iodide (PI) assay. Flow cytometric results reveal that the pretreatment of Molt4 cells with 6 mM NAC resulted in the inhibition of heteronemin-induced apoptosis by 39.3% at 0.15 μg/mL (Figure 5A). To verify the cause of this effect, Western blotting was used to study the expression of proapoptotic proteins including caspases-3, -8, -9 as well as γ-H2AX and PARP. The pretreatment of Molt4 cells with NAC (6 mM) significantly suppressed the expression of proapoptotic proteins induced by heteronemin (Figure 5B). Therefore, we postulated that the induction of ROS production by heteronemin significantly contributed to mitochondrial dysfunction and cell apoptosis. To test this hypothesis, we examined the effect of NAC pretreatment on MMP disruption caused by heteronemin (0.15 μg/mL). As shown in Figure 5C, NAC pretreatment significantly inhibited MMP disruption by 38.7%. To further examine whether the mitochondrial function was inhibited by heteronemin, we evaluated ATP production using ATP determination kit. As shown in Figure 5D, the mean luciferase value of Molt4 cells decreased from 3176 (control) to 2332 by heteronemin (0.15 μg/mL) after 24 h. The pretreatment of heteronemin-treated cells with NAC, restored the ATP production level (Figure 5D). These results suggest that heteronemin activated proapoptotic proteins, induced ROS overproduction by mitochondria resulting in mitochondrial dysfunction and apoptotic cell death.

### 2.5. Proteomic Analysis of Molt4 Treated Cells with Heteronemin

**Figure 6:**
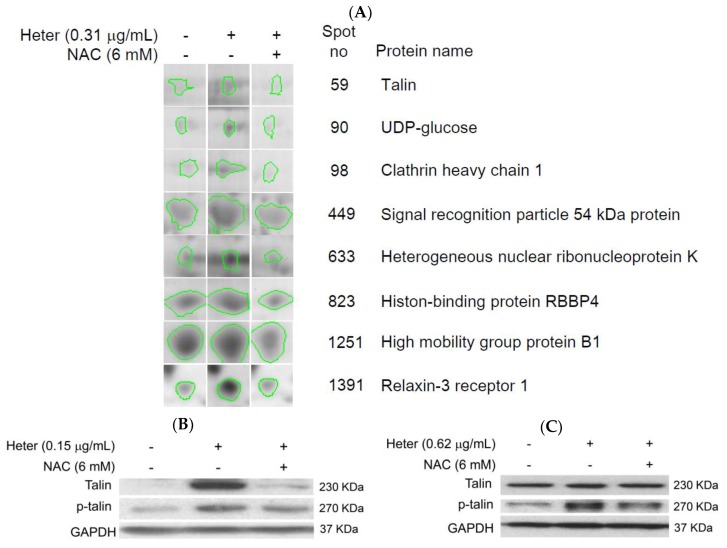

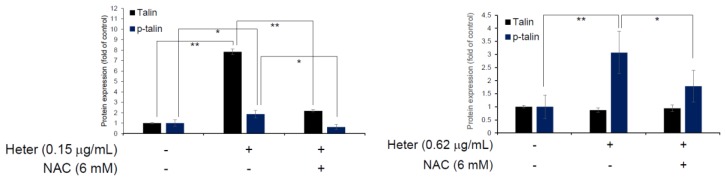
The differences in the expression of ROS-related proteins in three groups including the control, heteronemin, and combination (NAC + heteronemin) groups; (**A**) 2-D Gel electrophoresis showing the downregulation of ROS-associated proteins in the combination group (NAC + heteronemin) compared with heteronemin group in Molt4 cells; (**B**) Western blotting analysis comparing the difference in the expression of talin and p-talin in heteronemin group and in NAC + heteronemin group in Molt4 cells, GAPDH was used as the loading control; Results are presented as mean ± SD of three independent experiments (* *p* < 0.05; ** *p* < 0.01); (**C**) Western blotting analysis comparing the difference in the expression of talin and p-talin in heteronemin group and NAC + heteronemin group in HEK293 cells, GAPDH was used as the loading control. Results are presented as mean ± SD of three independent experiments (** *p* < 0.01).

**Figure 7:**
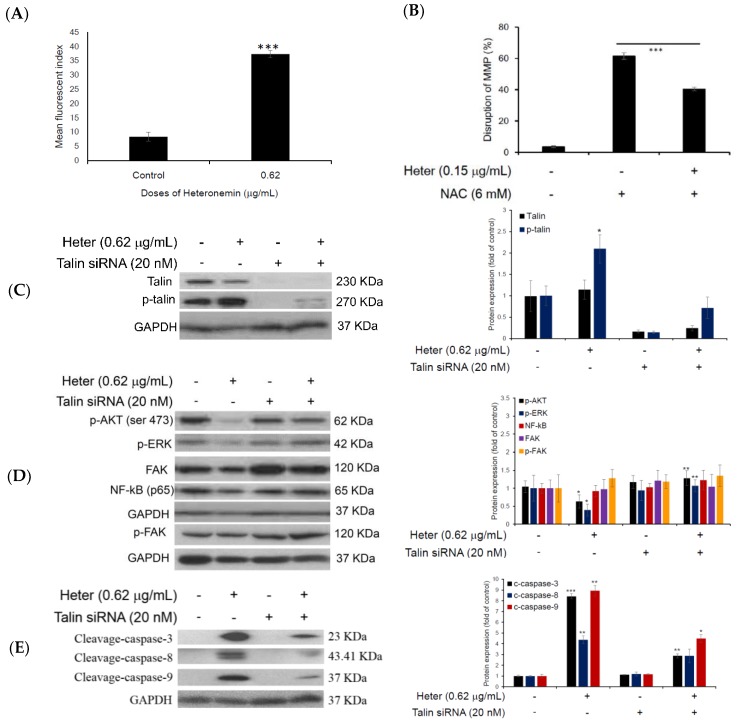

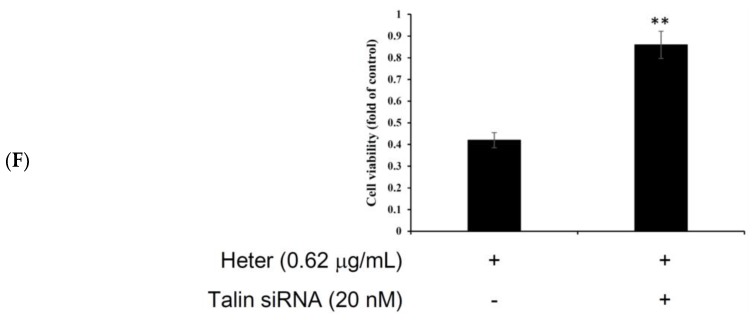
Cytotoxic mechanism of heteronemin determined in HEK293 cells. (**A**) Quantitative results of mitochondrial superoxide production. Results are presented as mean ± SD of three independent experiments, (*** *p* < 0.001); (**B**) Quantitative results of mitochondrial membrane potential; Results are presented as mean ± SD of three independent experiments, (*** *p* < 0.001); (**C**) Western blotting analysis comparing the difference in expression of talin and p-talin in heteronemin group and siRNA + heteronemin group; GAPDH was used as the loading control; Results are presented as mean ± SD of three independent experiments, (* *p* < 0.05); (**D**) Talin regulated FAK signaling pathway proteins including p-AKT (ser473), NF-κB (p65) and p-ERK in HEK293 cells; GAPDH was used as the loading control; Results are presented as mean ± SD of three independent experiments (* *p* < 0.05; ** *p* < 0.01); (**E**) Pro-apoptotic proteins were determined; GAPDH was used as the loading control; Results are presented as mean ± SD of three independent experiments (** *p* < 0.01; *** *p* < 0.001); (**F**) Cytotoxic effect of heteronemin after talin siRNA pretreatment; Results are presented as mean ± SD of three independent experiments (** *p* < 0.01).

To further clarify the underlying mechanism of heteronemin-induced Molt4 cells apoptosis, proteomic analysis was used to identify target proteins which promoted apoptotic cell death. Molt4 cells were treated with either DMSO or NAC followed by heteronemin for 2 h. LC-MS/MS analysis and the database search showed that 36 proteins were changed in heteronemin-treated cells compared with the control. Interestingly, the expression of 7 proteins was up-regulated (Table S1) while the expression of 11 proteins was down-regulated after heteronemin treatment (Table S2). The effect was suppressed with the pretreatment of NAC (Figure 6A). Interestingly, talin expression was upregulated after heteronemin treatment suggesting that talin may be an important target for heteronemin-induced apoptosis. To verify this hypothesis, we also determined talin and phosphorylated talin expressions using Western blotting analysis. In Figure 6B,C, the Western blotting results show that both talin and phosphorylated talin expressions were upregulated in Molt4 cells. In the HEK cell, only phosphorylated talin expression was upregulated. The pretreatment with NAC for 2 h, inhibited the upregulation of talin and phosphorylated talin expressions (Figure 6B,C). We also determined ROS generation and MMP disruption after heteronemin 0.62 μg/mL treatment for 24 h, in HEK293 cells. Our results reveal that heteronemin induced ROS generation by 4.5-folds compared with the control and 61% cells showed MMP disruption. NAC pretreatment restored the percentage of cells with disrupted MMP to 41% (Figure 7A,B). These results confirm that heteronemin induced ROS generation which upregulated the expression of talin phosphorylation.

To further evaluate the effect of heteronemin on the expression of phosphorylated talin and its downstream proteins, we knocked down talin expression by the transfection of siRNA specifically targeting the corresponding enzymes and evaluated the expression of the downstream proteins in HEK293 cells instead of Molt4 cells due to the low transfection efficiency in these cells. Western blotting analyses showed that the phosphorylation of talin was up-regulated after heteronemin treatment compared with the control group and talin siRNA significantly inhibited the activation of talin phosphorylation (Figure 7C). We also determined focal adhesion kinase (FAK) downstream proteins which are related to cells survival and proliferation. Our results confirm that heteronemin suppressed the expression of phosphorylated AKT and ERK but not FAK and NF-κB (p65), although the down-regulation was resumed with the transfection of talin siRNA, but the expression was not changed upon transfection of talin siRNA only (Figure 7D). These results demonstrate that heteronemin directly regulated talin phosphorylation expression which resulted in cell apoptosis, but it did not affect talin/FAK complex.

**Figure 8:**
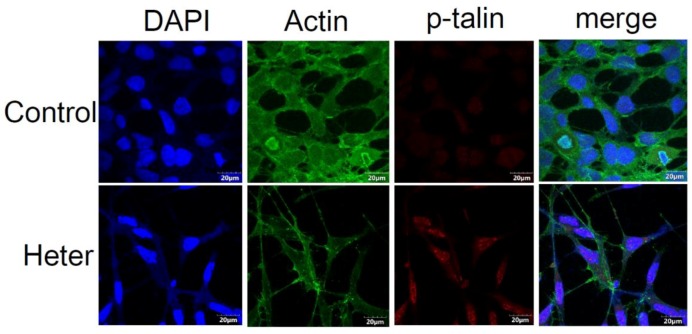
The alterations of p-talin and actin of HEK 293 cells after heteronemin 0.62 μg/mL treatment. The actin microfilaments showed spindle-like cell extensions. Confocal microscopy images of actin (green) and p-talin (red) stained HEK 293 cells. Cells were counterstained with DAPI to label the cell nuclei (blue).

To further evaluate whether phosphorylated talin plays a significant role in heteronemin-induced cell apoptosis, Western blotting was performed to detect cleaved caspases 3, 8 and 9. Heteronemin induced cleavage of caspases 3, 8 and 9 but the quantitative results show only that caspases 3 and 9 but not 8 were restored after transfection with talin siRNA. From this finding, we think that phosphorylated talin plays an important role in the intrinsic pathway which induces cell apoptosis following transfection with talin siRNA (Figure 7E). In addition, cell viability was also determined after heteronemin treatment and the transfection of talin siRNA. Our results show that the transfection of talin siRNA resumed the cytotoxic effect of heteronemin from 42% ± 0.03 to 86% ± 0.06 (Figure 7F). To evaluate the distribution of phosphorylated talin and cytoskeletal changes (actin), HEK293 cells were cultured with or without heteronemin 0.62 μg/mL for 24 h and the effect was determined using confocal microscopy (Figure 8). Interestingly, heteronemin treatment increased the distribution of phosphorylated talin, both in the cytosol and nuclear fraction. Heteronemin also changed the shape of actin to spindle-like cell extensions as well as it reduced the smoothness of the cell membrane as shown in Figure 8. These results confirm that heteronemin-induced cell apoptosis due to the upregulation of talin phosphorylation. The changes in actin microfilaments may also play a role in cellular apoptosis.

## 3. Discussion

Apoptosis refers to programmed cell death showing unique morphological changes including cell shrinkage, chromosomes condensation, DNA fragmentation, the formation of apoptotic bodies and exposure of phosphatidylserine on the cell surface [23]. These processes stimulate the removal of damaged cells by macrophages to avoid allergic or immunological reactions [24,25]. Apoptosis can be induced by two pathways, extrinsic or intrinsic [26]. In the extrinsic pathway, cell apoptosis is induced through first apoptosis signal receptor (Fas) binding to Fas ligand leading to the formation of the death-inducing signaling complex (DISC) which activates caspase-8 and the downstream caspase-3 [27]. On the other hand, the intrinsic pathway is related to mitochondria, which involves the induction of Bcl-2 family proteins, BAX expression and the release of cytochrome *c* in the cytoplasm which binds with APAF-1 and caspase-9 to form apoptosome leading to caspase-3 activation and the induction of cell apoptosis. Many of the recently developed anticancer agents focus on cell apoptosis due to the important role of this process in reversing drug resistance and tumor formation [28].

Nature especially marine environment proved to be an invaluable source of potent apoptotic agents such as alkylpyridinim salts, bryostatin-1, bromovulone III, fucoxanthinol, and onnamide A [29]. Sponge-derived secondary metabolites demonstrated potent cytotoxic potential which can be developed to successful anticancer agents due to their remarkable chemical diversity [30]. However, the limited supply of these bioactive compounds halts drug development initiatives. Our results show that heteronemin was the major compound in *Hippospongia* sp. that exhibited potent cytotoxic activity against several cancer cell lines suggesting its potential application as an antitumor agent. In a previous study, heteronemin induced human renal carcinoma cells A498 apoptosis through intrinsic pathway mediated by the disruption of mitochondrial membrane potential and induction of autophagy through P38 activation [31]. In prostate cancer cells and chronic myelogenous leukemia cells, the cytotoxic results indicate that heteronemin induced both the intrinsic and extrinsic pathways and cell apoptosis was mediated by Met/STAT3 pathway in prostate cancer [32] and NF-κB pathway in chronic myelogenous leukemia cells [22]. In this study, we demonstrated that heteronemin induced both the intrinsic and extrinsic pathways in acute lymphoblastic leukemia cells. The intrinsic pathway may serve as a major molecular target for heteronemin that acted as a potent apoptosis inducer.

Accumulating evidence suggested that mitochondria produce significant quantities of cellular ROS [33,34]. Several classes of natural products target mitochondria resulting in cell death such us curcumin, colchicine, paclitaxel and vinca alkaloid derivatives (SK228) [35,36,37,38]. In many types of cancer cells, elevated levels of ROS were detected due to the activation of their oncogenes, disturbance of mitochondrial functions compared with normal cells [39]. Exogenous agents also play a role in the generation of ROS rendering cancer cells more vulnerable to damage due to their unique metabolic and genetic features [40]. From our previous study, marine derived sesterterpenoids from *Carteriospongia* sp. promoted ROS generation and mediated cell apoptosis through HSP90 inhibition [41]. Such results encourage us to investigate whether heteronemin, a sesterterpenoid derivative, might induce oxidative stress in cancer cells. In this report, we demonstrated that heteronemin significantly increased ROS production from mitochondria. Pretreating Molt4 cells with NAC significantly protected the cells from oxidative stress induced by heteronemin. NAC suppressed the disruption of mitochondrial membrane potential. It also promoted ATP production and inhibited cell death. These results indicate that the cytotoxic activity of heteronemin depends on oxidative stress resulting in mitochondrial dysfunction. ROS trigger many signaling pathways in cancer cells to affect cell growth, differentiation, protein synthesis, glucose metabolism and survival pathways including MAPK/ERK, PI3K/Akt and IKK/NF-κB signaling pathways [42,43]. However, there is limited information on the relation between ROS generation and talin phosphorylation. We found that heteronemin upregulated the expression of phosphorylated talin through ROS production. The pretreatment of Molt4 and HEK293 cells with NAC resumed heteronemin-induced modulation of phosphorylated talin suggesting that talin phosphorylation might be a downstream molecule of ROS production.

Recent reports indicated that talin expression was higher in cancer cells, indicating that this protein is correlated with the oncogenic transformation process and may serve as an appealing therapeutic target as well as a prognostic marker [44,45,46,47,48]. Talin, which plays a significant role in integrin activation, promotes high-affinity interactions with extracellular matrix (ECM) proteins, affecting the outward conformational changes. It also recruits and activates FAK to form adhesion complexes which serve as bidirectional proteins for extracellular and intracellular signals [49]. The activation of FAK promotes the downstream signaling pathway AKT/NF-κB, resulting in the promotion of angiogenesis, cell survival and growth [12,50,51]. FAK also activates ERK which promotes survival advantage of tumor cells through improving their migratory and invasive properties [12,50]. However, some studies also demonstrated that the high expression of talin can decrease the ability of cells invasion and migration. It also decreases tumor grade in human liver cancer cell lines [10]. We observed that heteronemin treatment significantly upregulated the expression of talin and phosphorylated talin in Molt4 cells as well as upregulated phosphorylated talin in HEK293 cells. On the other hand, heteronemin suppressed the expression of phosphorylated AKT and ERK but not FAK and NF-κB (p65). In a previous study, it was demonstrated that the expression of AKT and ERK phosphorylation was inhibited after heteronemin treatment in human renal carcinoma cells, but the molecular mechanism was not further explored [31]. Our results indicate that the suppression of the expression of phosphorylated AKT and ERK were modulated by phosphorylated talin in Motl4 cells. Previous research indicated that talin can upregulate phosphorylated FAK and form talin/FAK complex to up-regulate p-AKT, ERK and NF-κB expression thus mediates cell proliferation. However, our results indicate that phosphorylated talin was up-regulated after treatment with heteronemin, and the expression of phosphorylated FAK did not change after heteronemin treatment. From this finding, we think phosphorylated talin may serve as a bifunctional protein and the anti-cancer effect of phosphorylated talin was not through the interaction with FAK. Our Western blotting assay also revealed that the pretreatment with talin siRNA suppressed the effect of heteronemin on pro-apoptotic proteins including cleaved caspases-3 and -9 but it did not affect DNA damage related protein, PARP (data not show). Our results suggest that the intrinsic pathway is the major signaling pathway in cell apoptosis mediated by talin phosphorylation. Cell viability results also confirm that phosphorylated talin is the target protein in heteronemin induced cell apoptosis. Interestingly, the phosphorylation of talin can interfere with actin microfilament which can lead to morphological changes [52].

## 4. Materials and Methods

### 4.1. Bioassay Materials

Cell lines were purchased from the American Type Culture Collection (Manassas, VA, USA). RPMI 1640 medium, fetal bovine serum (FBS), trypan blue, anti-anti, and penicillin G were purchased from Gibco (Gaithersburg, MD, USA)., tetrazolium bromide (MTT), dimethyl sulfoxide (DMSO), and all other chemicals were purchased from Sigma-Aldrich (St. Louis, MO, USA). Antibodies against cleaved-caspases-3 (1:250; Rabbit IgG), -8 (1:250; Mouse IgG), and -9 (1:250; Rabbit IgG), γ-H2AX (1:500; Rabbit IgG), cleaved-PARP (1:1000; Rabbit IgG) and phosphorylated talin (1:250; Rabbit IgG) were obtained from Cell Signaling Technologies (Beverly, MA, USA). Antibodies of talin (1:1000; Goat IgG), NF-κB (p65) (1:500; Rabbit IgG), FAK (1:500; Rabbit IgG), phosphorylated FAK (1:500; Rabbit IgG), AKT (ser473) (1:500; Rabbit IgG) and ERK (1:500; Mouse IgG) were obtained from Santa Cruz Biotechnology (Santa Cruz, CA, USA). Anti-mouse, rabbit and goat IgG secondary antibodies were obtained from Pierce (Rockford, IL, USA). Transfer membrane and ECL Western blotting substrate were purchased from Life Sciences (Amersham, UK). JC-1 for mitochondrial membrane potential and MitoSOX red for mitochondrial superoxide indicator were purchased from Molecular Probes (Carlsbad, CA, USA). The oxidative stress indicator (carboxy-H2DCFDA) was obtained from Invitrogen Detection Technologies (Carlsbad, CA, USA). The annexin-V-FITC/PI kit was purchased from Strong Biotech Corporation (Taipei, Taiwan).

### 4.2. Characterization of Heteronemin from Sponge Hippospongia sp.

Samples were collected by scuba diving at a depth of 20 m from the coral reefs of Tai-tung coast, Taiwan and freeze-dried and further extracted with EtOAc. Heteronemin was separated by silica gel column with *n*-hexane–EtOAc (3:1) [20]. An LC-20A VP HPLC system (Shimadzu Inc., Tokyo, Japan) was used for analysis equipped with a quaternary pump (LC-20AT), an on-line degasser (DGU-14A), a photodiode-array detector (SPD-M20A), an autosampler (SIL-20AD) and data collection using Class VP. Cosmosil 5C-18-MS-II column (5 μm, 150 × 4.6 mm I.D.) supplied by Nacalai Tesque, Inc. (Kyoto, Japan) used for liquid chromatography. Sample (10 μL) was injected and the mobile phase consisted of water (A) and ACN (B). Using gradient program as follows: the initial elution condition was A:B (25:75, *v*/*v*), linearly changed to A:B (12:88, *v*/*v*) at 10 min, A:B (4:96, *v*/*v*) at 15 min. The percentage of mobile phase B increased linearly to 100% within 15 min and 210 nm was selected as the detection wavelength. HPLC grade methanol (1 mL) was used to dissolve 1 mg from the *Hippospongia* sp. dry extract and filtered with 0.45 μm membrane filter before loading on the HPLC column, [53].

### 4.3. MTT Antiproliferative Assay

Culture plates (96-well) were used for the MTT assay. After seeding the cells at 4 × 10^4^ per well they were treated with several concentrations of the tested compounds. The cytotoxic effect of heteronemin was evaluated by MTT cell proliferation assay (thiazolyl blue tetrazolium bromide, Sigma-M2128) [54]. To measure light absorbance values, ELISA reader (Anthoslabtec Instrument, Salzburg, Austria) was used at 570 and 620 nm (OD = OD_570_ − OD_620_). Calculations were performed to determine the concentration that caused 50% inhibition (IC_50_). MTT assay results were expressed as a percentage of the control ± SD obtained from *n* = 4 wells per experiment from three independent experiments.

### 4.4. DNA Fragmentation Assay

Cells were seeded at 1 × 10^6^ in 10 cm dish before the treatment with different concentrations of heteronemin (0.15–0.62 μg/mL) or 0.1% DMSO for 24 h. Cold lysis buffer (containing 50 mM Tris-HCl, pH 7.5, 10 mM EDTA, 0.3% Triton X-100) was used to induce lysis in collected cells. Cells were incubated on ice for 30 min and centrifuged. The supernatant containing RNAase (100 ug/mL) was incubated at 50 °C for 30 min, then proteinase K (200 μg/mL) was add and incubated at 37 °C for 1 h. Phenol/chloroform mixture was used to extract DNA and it was precipitated with ethanol [55,56]. The extracted DNA was electrophoresed on a 2% agarose gel containing 0.1 μg/mL of ethidium bromide.

### 4.5. Annexin V/PI Apoptosis Assay

Cells were seeded at 1 × 10^6^ in the 10 cm dish before the treatment with different concentrations of heteronemin (0.07–0.31 μg/mL) or 0.1% DMSO for 24 h. The inner membrane phosphatidylserine (PS) quantified using an annexin V-FITC staining kit for the detection of cell apoptosis [57]. Cells (1 × 10^6^) were collected in eppendorf and labeled with annexin V-FITC (10 μg/mL) and PI (50 μg/mL) in 100 μL binding buffer. After labeling, the binding buffer (500–1000 μL) was added at a concentration of 2 × 10^5^ cells/mL before detection using a flow cytometer (Beckman Coulter, Taipei, Taiwan) and analyzed with WinMDI software.

### 4.6. Scanning Electron Microscopy

Cells were seeded at 1 × 10^6^ in 10 cm dish before the treatment with different concentrations of heteronemin (0.15–0.31 μg/mL) or 0.1% DMSO for 24 h. Cells surface changes were examined by scanning electron microscopy using the reported method [58]. Cells were dehydrated gradually with 30%, 50%, 70%, 80%, 90%, 95% and 100% ethanol (thrice) for 15 min (for each treatment) at room temperature and the cells were then air dried. Dried cells fixed on a specimen mount were sputter-coated with platinum (Ion sputter E-1010, Hitachi Inc., Tokyo, Japan) for 20 s and examined by an SEM (S-3500N, Hitachi Inc., Tokyo, Japan) operating at 15 kV. Energy dispersive spectrometer (EDS) was also used to analyze the interesting regions.

### 4.7. Determination of ROS Generation, Mitochondrial Superoxide Production and MMP Disruption

Mitochondrial membrane potential disruption was detected with JC-1 dye (5 μg/mL), which binds to mitochondria and forms a monomer when membrane potential was down-regulated. Mitochondrial superoxide production was detected with MitoSOX Red (5 mM; Molecular Probes) and carboxy-H2DCFDA (1 mM) was used to detect ROS generation [57]. Cells were labeled with fluorescent dye for 30 min then washed with PBS before analysis by flow cytometry.

### 4.8. Western Blotting Analysis

Ice-cold RIPA lysis buffer was added to cell lysates for 30 min. The lysates were centrifuged at 18,000 rpm for 30 min, the supernatant was separated to determine protein concentration by BCA protein assay kit (Rockford, IL, USA). 20 μL of proteins were separated by 8%, 10% or 15% of SDS gel electrophoresis and transferred using PVDF membrane. The transferred membrane was blocked with 5% nonfat dry milk tTBS buffer for 1 h. Protein expression was monitored by immunoblotting using specific antibodies. Proteins expressions on PVDF membrane were detected by chemiluminescence kit (Rockford, IL, USA) [59].

### 4.9. ATP Determination

ATP determination was achieved following the manufacturer’s protocol (Invitrogen™ cat: A22066, Carlsbad, CA, USA). After treatment with heteronemin (0.15 μg/mL), cells were lysed with RIPA lysis buffer and centrifuged at 18,000 rpm for 30 min, the supernatant was separated to determine protein concentration by BCA protein assay kit. A reconstituted buffer was used containing dH_2_O, 20× Reaction Buffer, 0.1 M DTT, 10 mM d-luciferin, 12.5 ng firefly luciferase solution and cell lysate. Luminescence was analyzed with H4 Hybrid Multi-Mode Microplate Reader (BioTek, Winooski, VT, USA).

### 4.10. Animal Xenograft Model

Six-week-old male Balb/c nude mice were obtained from the National Laboratory Animal and Research Center (Taipei, Taiwan). 0.2 mL PBS was used to resuspend Molt4 cells (2 × 10^6^) injected subcutaneously into the right flank of each mouse. Fourteen days after tumor cell injection, tumor growths were confirmed and randomly divided into two groups (each group contained six mice). Heteronemin (0.31 μg/g) was intraperitoneally administered to the treatment group and the control group which received solvent (DMSO) only. Heteronemin was administrated three times a week for 24 days each time injected 100 μL/mice. Carbon dioxide were used to sacrifice animals. Calipers was used to measure tumor spherical size three times a week and the tumor volumes were calculated according to the standard formula: width^2^ × length/2. All applicable international, national, and/or institutional guidelines for the care and use of animals were followed [41]. 

### 4.11. Immunofluorescence Analysis

After treatment with heteronemin (0.62 μg/mL), 2% paraformaldehyde was used to fix cells for 30 min and 0.2% Trition X-100 in PBS was used for the permeabilization for 20 min [54]. 5% BSA and 0.05% Trition X-100 (T-PBS) were used to prevent non-specific protein binding for 1 h. Cells were then incubated with the phosphorylated talin and actin (1:250) for 2 h and the secondary antibodies for 1 h for 1:1000 (Alexa Fluor 586-conjugated goat anti-mouse IgG or Alexa Fluor 488-conjugated goat anti-rabbit IgG, Life Technologies, Carlsbad, CA, USA). After washing with PBS, cells were monitored with FV1000 confocal laser scanning microscope (Olympus, Tokyo, Japan).

### 4.12. Two-Dimensional Gel Electrophoresis

Cells were seeded at 1 × 10^6^ in the 10-cm dish before the treatment with 0.31 μg/mL of heteronemin or 0.1% DMSO for 2 h. After harvesting, cold acetone was added at −20 °C for at least 30 min. After centrifugation of the precipitate at 12,000 rpm for 10 min at 4 °C., cold acetone with 20 mM DTT was used to wash the precipitate then air dried. Protein pellets were dissolved in rehydration buffer (7M urea, 2M thiourea, 2% CHAPS, 0.5% IPG buffer, 20 mM DTT). Bradford method was used to measure protein concentration. Immobiline DryStrips (13 cm) (pH 3–10) were rehydrated for 12 h at room temperature in 250 μL rehydration buffer. 100 μL sample buffer and a trace of bromophenol blue was mixed with 150 μg protein. The following IEF parameters was used to achieve protein focusing: 350 V, step and hold, 3 h; 650 V, gradient, 1 h; 1100 V, gradient, 1 h; 8000 V, gradient, 1.5 h; 8000 V, step and hold, 3 h, giving a total of 14,500 Vh. After IEF analysis, strips were removed for second-dimensional electrophoresis. The DryStrips were shaken in equilibration buffer for 15 min then the buffer was changed to another buffer containing 2.5% *w*/*v* iodoacetamide for 15 min. The second dimension was used SDS-PAGE (12% *w*/*v*). Silver staining was used to visualize the gel with Image Master 2D Platinum [60] The reported results were from three independent experiments using the same experimental condition.

### 4.13. LC-MS/MS Analysis and Database Searching

After in-gel digestion, the peptide mixture was separated by a Surveyor HPLC system (Thermo Finnigan, Carlsbad, CA, USA) utilizing a 75 μm i.d. capillary column packed with 5-mm particles (Bio-Red, California, USA) and then subjected to mass analysis using LCQ DECA XP MAX ion trap mass spectrometer (Thermo Finnigan, CA, USA) equipped with a nano-ESI source (Thermo Finnigan). All MS/MS were searched using MASCOT Distiller (Matrix Science, Boston, UK) and the resulting MGF file was searched using the Mascot search engine v2.2 (Matrix Science, Boston, UK). The search parameters were set as: Swiss-Prot; Homo sapiens (human); one trypsin missed cleavage was allowed; the mass tolerance was set at 2 Da for the precursor and 1 Da for the produced ions; carbamidomethyl was chosen for fixed modification; oxidation and deamidated were chosen for variable modifications; proteins with scores above the significance threshold (*p* < 0.05) were shown as significant hits.

### 4.14. Small Interfering RNA (siRNA)-Specific Targeted Gene and Cell Transfection

Talin siRNA sequences were purchased from Ambion (Thermo Fisher Scientific, Inc.). HEK293 were seeded at 6-well plates (2 × 10^5^ cells/well) and cultured in 2 mL medium containing 10% FBS. Cells were transfected with siRNA (20 nM) or the scramble control siRNA, using Lipofectamine 3000 (Invitrogen; Thermo Fisher Scientific, Inc.). Subsequently, the transfected cells were treated with heteronemin for 24 h.

### 4.15. Statistics

These results were expressed as mean ± SD. Comparison in each experiment was performed by Student’s *t*-test and Two-Way ANOVA for MTT results. *p* < 0.05 was considered significant.

## 5. Conclusions

We found that the treatment of Molt4 cells with heteronemin induced mitochondrial dysfunction and mitochondrial oxidative stress, leading to apoptosis. In vivo animal model further supported that heteronemin can act as potential antitumor agent. Our results also indicate that the treatment with heteronemin upregulated the expression of phosphorylated talin in Molt4 and HEK293 cells. The upregulation of phosphorylated talin interfered with actin microfilament and caused spindle-like shape cell extension. Our results provide a further correlation between ROS generation and phosphorylated talin activation and their effect on cancer cell death. Secondary metabolites targeting these processes represent interesting antileukemic agents.

## Supplementary Materials

The following are available online at http://www.mdpi.com/1660-3397/16/6/212/s1, Table S1: Identification of upregulation of ROS-associated proteins compared with heteroneim treatment in Molt 4 cells, Table S2: Identification of downregulation of ROS-associated proteins compared with heteroneim treatment in Molt 4 cells.

## References

[1] Skulachev V.P. (2006). Bioenergetic aspects of apoptosis, necrosis and mitoptosis. Apoptosis.

[2] Yang Y., Liu H., Liu F., Dong Z. (2014). Mitochondrial dysregulation and protection in cisplatin nephrotoxicity. Arch. Toxicol..

[3] Weinberg S.E., Chandel N.S. (2015). Targeting mitochondria metabolism for cancer therapy. Nat. Chem. Biol..

[4] Dikalov S. (2011). Cross talk between mitochondria and NADPH oxidases. Free Radic. Biol. Med..

[5] Hosoki A., Yonekura S., Zhao Q.L., Wei Z.L., Takasaki I., Tabuchi Y., Wang L.L., Hasuike S., Nomura T., Tachibana A. (2012). Mitochondria-targeted superoxide dismutase (SOD2) regulates radiation resistance and radiation stress response in HeLa cells. J. Radiat. Res..

[6] Panieri E., Santoro M.M. (2016). ROS homeostasis and metabolism: A dangerous liason in cancer cells. Cell Death Dis..

[7] Zorov D.B., Juhaszova M., Sollott S.J. (2014). Mitochondrial reactive oxygen species (ROS) and ROS-induced ROS release. Physiol. Rev..

[8] Sabharwal S.S., Schumacker P.T. (2014). Mitochondrial ROS in cancer: Initiators, amplifiers or an Achilles’ heel?. Nat. Rev. Cancer.

[9] Morgan M.J., Liu Z.G. (2011). Crosstalk of reactive oxygen species and NF-kappa B signaling. Cell Res..

[10] Fang K.P., Zhang J.L., Ren Y.H., Qian Y.B. (2014). Talin-1 correlates with reduced invasion and migration in human hepatocellular carcinoma cells. Asian Pac. J. Cancer Prev..

[11] Santaclara F., Lago J., Vieites J.M., Cabado A.G. (2005). Effect of okadaic acid on integrins and structural proteins in BE(2)-M17 cells. Arch. Toxicol..

[12] Sulzmaier F.J., Jean C., Schlaepfer D.D. (2014). FAK in cancer: Mechanistic findings and clinical applications. Nat. Rev. Cancer.

[13] Goswami A., Shah B.A., Kumar A., Rizvi M.A., Kumar S., Bhushan S., Malik F.A., Batra N., Joshi A., Singh J. (2014). Antiproliferative potential of a novel parthenin analog P16 as evident by apoptosis accompanied by down-regulation of PI3K/AKT and ERK pathways in human acute lymphoblastic leukemia MOLT-4 cells. Chem. Biol. Interact..

[14] Cheng J.Q., Lindsley C.W., Cheng G.Z., Yang H., Nicosia S.V. (2005). The Akt/PKB pathway: Molecular target for cancer drug discovery. Oncogene.

[15] Xing B., Thuppal S., Jedsadayanmata A., Du X., Lam S.C. (2006). TA205, an anti-talin monoclonal antibody, inhibits integrin-talin interaction. FEBS Lett..

[16] Zhang W., Mao Y.Q., Wang H., Yin W.J., Zhu S.X., Wang W.C. (2015). MiR-124 suppresses cell motility and adhesion by targeting talin 1 in prostate cancer cells. Cancer Cell Int..

[17] Chen P., Zheng X., Zhou Y., Xu Y., Zhu L., Qian Y. (2017). Talin-1 interaction network promotes hepatocellular carcinoma progression. Oncotarget.

[18] Watters D., Garrone B., Gobert G., Williams S., Gardiner R., Lavin M. (1996). Bistratene A causes phosphorylation of talin and redistribution of actin microfilaments in fibroblasts: Possible role for PKC-delta. Exp. Cell Res..

[19] Zhang C., Liu Y. (2015). Targeting cancer with sesterterpenoids: The new potential antitumor drugs. J. Nat. Med..

[20] Chang Y.C., Tseng S.W., Liu L.L., Chou Y., Ho Y.S., Lu M.C., Su J.H. (2012). Cytotoxic sesterterpenoids from a sponge *Hippospongia* sp.. Mar. Drugs.

[21] Cassiano C., Esposito R., Tosco A., Zampella A., D’Auria M.V., Riccio R., Casapullo A., Monti M.C. (2014). Heteronemin, a marine sponge terpenoid, targets TDP-43, a key factor in several neurodegenerative disorders. Chem. Commun. (Camb).

[22] Schumacher M., Cerella C., Eifes S., Chateauvieux S., Morceau F., Jaspars M., Dicato M., Diederich M. (2010). Heteronemin, a spongean sesterterpene, inhibits TNF alpha-induced NF-kappa B activation through proteasome inhibition and induces apoptotic cell death. Biochem. Pharmacol..

[23] Wong R.S. (2011). Apoptosis in cancer: From pathogenesis to treatment. J. Exp. Clin. Cancer Res..

[24] Kerr J.F., Wyllie A.H., Currie A.R. (1972). Apoptosis: A basic biological phenomenon with wide-ranging implications in tissue kinetics. Br. J. Cancer.

[25] Kurosaka K., Takahashi M., Watanabe N., Kobayashi Y. (2003). Silent cleanup of very early apoptotic cells by macrophages. J. Immunol..

[26] Ichim G., Tait S.W. (2016). A fate worse than death: Apoptosis as an oncogenic process. Nat. Rev. Cancer.

[27] Wilson N.S., Dixit V., Ashkenazi A. (2009). Death receptor signal transducers: Nodes of coordination in immune signaling networks. Nat. Immunol..

[28] Fulda S., Debatin K.M. (2006). Extrinsic versus intrinsic apoptosis pathways in anticancer chemotherapy. Oncogene.

[29] Montaser R., Luesch H. (2011). Marine natural products: A new wave of drugs?. Future Med. Chem..

[30] Anjum K., Abbas S.Q., Shah S.A., Akhter N., Batool S., Hassan S.S. (2016). Marine Sponges as a Drug Treasure. Biomol. Ther. (Seoul).

[31] Wu S.Y., Sung P.J., Chang Y.L., Pan S.L., Teng C.M. (2015). Heteronemin, a spongean sesterterpene, induces cell apoptosis and autophagy in human renal carcinoma cells. Biomed. Res. Int..

[32] Wu J.C., Wang C.T., Hung H.C., Wu W.J., Wu D.C., Chang M.C., Sung P.J., Chou Y.W., Wen Z.H., Tai M.H. (2016). Heteronemin is a novel c-Met/STAT3 inhibitor against advanced prostate cancer cells. Prostate.

[33] Starkov A.A. (2010). Measurement of mitochondrial ROS production. Methods Mol. Biol..

[34] Li X., Fang P., Mai J., Choi E.T., Wang H., Yang X.F. (2013). Targeting mitochondrial reactive oxygen species as novel therapy for inflammatory diseases and cancers. J. Hematol. Oncol..

[35] Trujillo J., Granados-Castro L.F., Zazueta C., Anderica-Romero A.C., Chirino Y.I., Pedraza-Chaverri J. (2014). Mitochondria as a target in the therapeutic properties of curcumin. Arch. Pharm..

[36] Urakami K., Zangiacomi V., Yamaguchi K., Kusuhara M. (2013). Impact of 2-deoxy-d-glucose on the target metabolome profile of a human endometrial cancer cell line. Biomed. Res..

[37] Urra F.A., Cordova-Delgado M., Pessoa-Mahana H., Ramirez-Rodriguez O., Weiss-Lopez B., Ferreira J., Araya-Maturana R. (2013). Mitochondria: A promising target for anticancer alkaloids. Curr. Top. Med. Chem..

[38] Huang S.M., Hsu P.C., Chen M.Y., Li W.S., More S.V., Lu K.T., Wang Y.C. (2012). The novel indole compound SK228 induces apoptosis and FAK/Paxillin disruption in tumor cell lines and inhibits growth of tumor graft in the nude mouse. Int. J. Cancer.

[39] Pelicano H., Carney D., Huang P. (2004). ROS stress in cancer cells and therapeutic implications. Drug Resist. Updat..

[40] Daga M., Ulllio C., Argenziano M., Dianzani C., Cavalli R., Trotta F., Ferretti C., Zara G.P., Gigliotti C.L., Ciamporcero E.S. (2016). GSH-targeted nanosponges increase doxorubicin-induced toxicity “in vitro” and “in vivo” in cancer cells with high antioxidant defenses. Free Radic. Biol. Med..

[41] Lai K.H., Liu Y.C., Su J.H., El-Shazly M., Wu C.F., Du Y.C., Hsu Y.M., Yang J.C., Weng M.K., Chou C.H. (2016). Antileukemic Scalarane Sesterterpenoids and Meroditerpenoid from *Carteriospongia* (*Phyllospongia*) sp., Induce Apoptosis via Dual Inhibitory Effects on Topoisomerase II and Hsp90. Sci. Rep..

[42] Liou G.Y., Storz P. (2010). Reactive oxygen species in cancer. Free Radic. Res..

[43] Storz P. (2005). Reactive oxygen species in tumor progression. Front. Biosci..

[44] Sakamoto S., McCann R.O., Dhir R., Kyprianou N. (2010). Talin1 promotes tumor invasion and metastasis via focal adhesion signaling and anoikis resistance. Cancer Res..

[45] Lai M.T., Hua C.H., Tsai M.H., Wan L., Lin Y.J., Chen C.M., Chiu I.W., Chan C., Tsai F.J., Jinn-Chyuan Sheu J. (2011). Talin-1 overexpression defines high risk for aggressive oral squamous cell carcinoma and promotes cancer metastasis. J. Pathol..

[46] Kanamori H., Kawakami T., Effendi K., Yamazaki K., Mori T., Ebinuma H., Masugi Y., Du W., Nagasaka K., Ogiwara A. (2011). Identification by differential tissue proteome analysis of talin-1 as a novel molecular marker of progression of hepatocellular carcinoma. Oncology.

[47] Sakamoto S., Kyprianou N. (2010). Targeting anoikis resistance in prostate cancer metastasis. Mol. Asp. Med..

[48] Xu Y.F., Ren X.Y., Li Y.Q., He Q.M., Tang X.R., Sun Y., Shao J.Y., Jia W.H., Kang T.B., Zeng M.S. (2015). High expression of Talin-1 is associated with poor prognosis in patients with nasopharyngeal carcinoma. BMC Cancer.

[49] Wang P., Ballestrem C., Streuli C.H. (2011). The C terminus of talin links integrins to cell cycle progression. J. Cell Biol..

[50] Desiniotis A., Kyprianou N. (2011). Significance of talin in cancer progression and metastasis. Int. Rev. Cell Mol. Biol..

[51] Zhang X., Tang N., Hadden T.J., Rishi A.K. (2011). Akt, FoxO and regulation of apoptosis. Biochim. Biophys. Acta.

[52] Brandhagen B.N., Tieszen C.R., Ulmer T.M., Tracy M.S., Goyeneche A.A., Telleria C.M. (2013). Cytostasis and morphological changes induced by mifepristone in human metastatic cancer cells involve cytoskeletal filamentous actin reorganization and impairment of cell adhesion dynamics. BMC Cancer.

[53] Wu T.-Y., Du Y.-C., Hsu Y.-M., Lu C.-Y., Singab A.N.B., El-Shazly M., Hwang T.-L., Lin W.-Y., Lai K.-H., Lu M.-C. (2013). New approach to the characterization and quantification of Antrodia cinnamomea benzenoid components utilizing HPLC-PDA, qNMR and HPLC-tandem MS: Comparing the wild fruiting bodies and its artificial cultivated commercial products. Food Res. Int..

[54] Shih H.C., El-Shazly M., Juan Y.S., Chang C.Y., Su J.H., Chen Y.C., Shih S.P., Chen H.M., Wu Y.C., Lu M.C. (2014). Cracking the cytotoxicity code: Apoptotic induction of 10-acetylirciformonin B is mediated through ROS generation and mitochondrial dysfunction. Mar. Drugs.

[55] Ray S.D., Sorge C.L., Raucy J.L., Corcoran G.B. (1990). Early loss of large genomic DNA in vivo with accumulation of Ca2+ in the nucleus during acetaminophen-induced liver injury. Toxicol. Appl. Pharmacol..

[56] Shen W., Kamendulis L.M., Ray S.D., Corcoran G.B. (1991). Acetaminophen-induced cytotoxicity in cultured mouse hepatocytes: Correlation of nuclear Ca2+ accumulation and early DNA fragmentation with cell death. Toxicol. Appl. Pharmacol..

[57] Su J.H., Chen Y.C., El-Shazly M., Du Y.C., Su C.W., Tsao C.W., Liu L.L., Chou Y., Chang W.B., Su Y.D. (2013). Towards the small and the beautiful: A small dibromotyrosine derivative from *Pseudoceratina* sp. sponge exhibits potent apoptotic effect through targeting IKK/NFkappaB signaling pathway. Mar. Drugs.

[58] Passey S., Pellegrin S., Mellor H. (2007). Scanning electron microscopy of cell surface morphology. Curr. Protoc. Cell Biol..

[59] Lu M.C., Du Y.C., Chuu J.J., Hwang S.L., Hsieh P.C., Hung C.S., Chang F.R., Wu Y.C. (2009). Active extracts of wild fruiting bodies of Antrodia camphorata (EEAC) induce leukemia HL 60 cells apoptosis partially through histone hypoacetylation and synergistically promote anticancer effect of trichostatin A. Arch. Toxicol..

[60] Chou C.H., Chuang L.Y., Lu C.Y., Guh J.Y. (2015). Vitamin D-binding protein is required for the protective effects of vitamin D in renal fibroblasts and is phosphorylated in diabetic rats. Mol. Cell Endocrinol..

