# Supplementary material for: Breaking down Leukemia Walls: Heteronemin, a Sesterterpene Derivative, Induces Apoptosis in Leukemia Molt4 Cells through Oxidative Stress, Mitochondrial Dysfunction and Induction of Talin Expression
Source: Mar Drugs. 2018 Jun 17;16(6):212. doi: 10.3390/md16060212 (PMC6025351; doi:10.3390/md16060212)
Supplement: Supplementary file 1 [file marinedrugs-16-00212-s001.pdf]

Supplementary Materials: Breaking Down Leukemia Walls: Heteronemin, a Sesterterpene Derivative, Induces Apoptosis in Leukemia Molt4 Cells through Oxidative Stress, Mitochondrial Dysfunction and Induction of Talin Expression

Table S1. Identification of upregulation of ROS-associated proteins compared with heteroneim treatment in Molt 4 cells.

Spot Number	Protein name	Spot expression (fold of control)	
		Heter	Heter+NAC
59	Talin-1	1.20 ±0.001	0.64 ±0.006
90	UDP-glucose	2.29 ±0.005	1.41 ±0.003
98	Clathrin heavy chain 1	1.72 ±0.017	1.20 ±0.012
633	Heterogeneous nuclear ribonucleoprotein K	2.99 ±0.107	0.67 ±0.018
823	Histone-binding protein RBBP4	1.09 ±0.060	0.76 ±0.034
1251	High mobility group protein B1	1.34 ±0.026	1.09 ±0.093
1391	Relaxin-3 receptor 1	1.29 ±0.340	0.57 ±0.121

Table S2. Identification of downregulation of ROS-associated proteins compared with heteroneim treatment in Molt 4 cells.

Spot Number	Protein name	Spot expression (fold of control)	
		Heter	Heter+NAC
680	T-complex protein 1 subunit epsilon	0.38±0.005	0.86 ±0.029
801	T-complex protein 1 subunit beta	0.40 ±0.024	1.15 ±0.064
965	Basic leucine zipper and W2 domain-containing protein 1	0.77 ±0.012	1.13 ±0.039
1231	Proteasome activator complex subunit 2	0.74 ±0.036	1.01 ±0.159
1253	6-phosphogluconolactonase	0.70 ±0.017	1.15 ±0.033
1302	Zinc finger protein 575	0.76 ±0.014	0.84 ±0.049
1311	Glutathione S-transferase P	0.83 ±0.053	1.05 ±0.106
1312	Thymidylate kinase	0.51 ±0.051	0.83 ±0.051
1324	Glucosamine 6-phosphate N-acetyltransferase	0.44 ±0.008	0.95 ±0.038
1331	ATP synthase subunit d, mitochondrial	0.80 ±0.004	1.28 ±0.038
1364	Nucleoside diphosphate kinase B	0.71 ±0.003	1.09 ±0.173

© 2018 by the authors. Submitted for possible open access publication under the terms and conditions of the Creative Commons Attribution (CC BY) license (http://creativecommons.org/licenses/by/4.0/).
